# Adapting the SLIM diabetes prevention intervention to a Dutch real-life setting: joint decision making by science and practice

**DOI:** 10.1186/1471-2458-13-457

**Published:** 2013-05-08

**Authors:** Sophia C Jansen, Annemien Haveman-Nies, Geerke Duijzer, Josien Ter Beek, Gerrit J Hiddink, Edith JM Feskens

**Affiliations:** 1 Community Health Service GGD Gelre-IJssel; Academic Collaborative Centre AGORA, GGD Gelre-IJssel, PO Box 51, 7300, AB, Apeldoorn, The Netherlands; 2 Division of Human Nutrition, Wageningen University; Academic Collaborative Centre AGORA, AGORA, The Netherlands; 3 Department of Social Sciences, Sub Department of Communication Sciences, Communication Strategies, Wageningen University, Wageningen, The Netherlands

**Keywords:** Diabetes, Prevention, Decision making, Evidence-based, Real-life setting, Practice, Adaptation

## Abstract

**Background:**

Although many evidence-based diabetes prevention interventions exist, they are not easily applicable in real-life settings. Moreover, there is a lack of examples which describe the adaptation process of these interventions to practice. In this paper we present an example of such an adaptation. We adapted the SLIM (Study on Lifestyle intervention and Impaired glucose tolerance Maastricht) diabetes prevention intervention to a Dutch real-life setting, in a joint decision making process of intervention developers and local health care professionals.

**Methods:**

We used 3 adaptation steps in accordance with current adaptation frameworks. In the first step, the elements of the SLIM intervention were identified. In the second step, these elements were judged for their applicability in a real-life setting. In the third step, adaptations were proposed and discussed for those elements which were deemed not applicable. Participants invited for this process included intervention developers and local health care professionals (n=19).

**Results:**

In the first adaptation step, a total of 22 intervention elements were identified. In the second step, 12 of these 22 intervention elements were judged as inapplicable. In the third step, a consensus was achieved for the adaptations of all 12 elements. The adapted elements were in the following categories: target population, techniques, intensity, delivery mode, materials, organisational structure, and political and financial conditions. The adaptations either lay in changing the SLIM protocol (6 elements) or the real-life working procedures (1 element), or a combination of both (4 elements).

**Conclusions:**

The positive result of this study is that a consensus was achieved within a relatively short time period (nine months) between the developers of the SLIM intervention and local health care professionals on the adaptations needed to make SLIM applicable in a Dutch real-life setting. Our example shows that it is possible to combine the perspectives of scientists and practitioners, and to find a balance between evidence-base and applicability concerns.

## Background

Worldwide several randomised controlled trials have shown that type 2 diabetes can be prevented by lifestyle interventions directed at diet and physical activity. The Finnish Diabetes Prevention Study and the US Diabetes Prevention Project for example, revealed a 58% risk reduction in the progression from impaired glucose tolerance to type 2 diabetes during a 3-year intervention [1,2]. In the Netherlands, a similar intervention study, the Study of Lifestyle intervention and Impaired glucose tolerance Maastricht (SLIM) has been undertaken. The SLIM intervention consisted of personal dietary advice, based on the Dutch guidelines for a healthy diet, during a 1-hour counselling session with a skilled dietician every 3 months. The objective of the intervention was to achieve a body weight loss of 5–7%. Moreover, subjects were encouraged to participate in a combined aerobic- and resistance exercise programme. Control subjects were only briefly informed about the beneficial effects of a healthy diet and physical activity, and received no individual advice [3] (Table 1).

**Table 1:** Details of the SLIM intervention

	
Dietary advice	Four times/year individual advice by dietician
	Based on Dutch dietary guidelines
	One group session/year
	Goal: 5–7% weight reduction
	No very-low calorie diet
Exercise training	Group sessions 1-2 hours/week by sports instructor
	Combined aerobic- and resistance exercise programme
	Individual advice on physical activity in daily life
	Goal: increase physical activity to at least 30 minutes/day for at least 5 days/week

The SLIM intervention was effective, evident in the 4-year cumulative diabetes incidence, which was 47% lower in the intervention group compared to the control group [4]. However, diabetes prevention interventions such as SLIM are not easily applicable in public health practice. SLIM was developed in a research setting in which interventions are often intensive, standardised and delivered by specially educated staff using strict protocols [5]. In this research setting, the process of deliverance is often isolated from a broader context in order to reduce the influence of confounding factors. Real-life settings, on the other hand, require more feasible and flexible interventions, compatible with the professional functioning of that setting. In real-life settings, interventions are delivered by varying staff members from diverse organisations, they are embedded in the societal and health care structure and are offered to heterogeneous populations [6]. Thus, research settings and real-life settings differ substantially. This implies the necessity of a translation process to adapt the intervention from science to practice before actual implementation can start.

When translating an intervention from science to practice, adaptations are inevitable due to the differences between research and real-life settings as described above. However, these adaptations may have consequences, since actual intervention effects are highly dependent on specific intervention elements such as target population, techniques, delivery mode, deliverers, intensity, materials and organisational structure [7]. When an intervention is adapted too much, it may not be effective anymore. Therefore, the challenge is to retain elements which are perceived essential for intervention effectiveness, while at the same time making the necessary adaptations to ensure the applicability in the local setting. The necessary balance is delicate and gives rise to much debate [8-11]. One of the problems is the difficulty of indicating which intervention elements are essential for intervention effectiveness [12]. Another problem is the question of responsibility: who decides which adaptations are necessary to make the intervention applicable in a real-life setting? Several authors have pointed out that adaptation decisions should be made collectively by both intervention developers and stakeholders in the local setting [13-15]. Remarkably, no examples of such joint decision making have been described so far.

The attention for adaptation issues is relatively new and has been increasing in the last decade. In 1995, Rogers defined adaptation as “the degree to which an innovation is changed or modified by a user in the process of its adoption and implementation” [16]. In 2001, Backer provided one of the first literature overviews on this topic [15]. In order to stimulate the gathering of research evidence, he proposed a six-step framework for the adaptation of interventions to real-life (“field”) settings, including: identifying the theory behind the intervention, conducting a component analysis of the intervention, assessing applicability concerns for the specific local setting, consulting with the intervention developers, consulting with the local setting, and finally, developing an implementation plan. McKleroy [14] proposed a slightly different five-step framework, including an assessment of the real-life setting and population, choosing whether or not to adapt the intervention, actually adapting the materials, pilot testing the adapted intervention, and finally, implementing it in the real-life setting. Several comparable frameworks have been proposed since then [13,17]. Bartholomew et al. recently added a new chapter to their book Intervention Mapping Techniques, making an argument for the added value of intervention mapping techniques during the adaptation process [18]. Across this literature, there is a persistent call for examples of studies documenting the different types of adaptations (deletions, additions, and modifications in content and changes in intensity), and the decisions leading to such adaptations, including their justification.

This study presents the example of the evidence-based SLIM intervention which was adapted to a Dutch real-life setting. Our aim was to identify which SLIM elements need adaptation in order to be implemented in practice, and to explore how to adapt these elements, in a joint decision making process of intervention developers and local health care professionals.

We believe that this study adds to current knowledge on adaptation issues. So far, there are few examples available in the field of diabetes prevention. Several randomised controlled trials have been translated to practice, however, only a few of these studies described the adaptations that were made to the original intervention, and they did not document the decision making process which lead to the adaptations. The Finnish Diabetes Prevention Study (DPS) has been translated to Finnish and Australian primary health care, community and workplace settings [19-22], but we are not aware of systematic reporting of adaptations to the DPS for implementation purposes. Also the US Diabetes Prevention Program (DPP) has been translated to a variety of settings. Jackson [23] summarises the adaptations which have been made to the DPP in seven different real-life variants, which include adaptations to e.g. delivery mode, intensity and patient materials. However, he concludes that six out of seven studies did not completely reveal the modifications they made to DPP. Several other translational studies of the DPP have since been published, but they tend to report adaptations to the DPP only shortly, without mentioning the decision making process [24-27]. Furthermore, the UK European Diabetes Prevention Study (EDPS) has been translated to a real-life community setting. The adaptations to the EDPS were described, but not the decision making process [28]. Our example is one of the first to elaborate on the joint decision making processes between researchers and practitioners in order to make evidence-based interventions suitable for implementation in real-life settings.

## Methods

In our study we made use of the adaptation frameworks as described in the Background [13-15,17,18]. We incorporated the steps which are essential to all frameworks: Step 1: Assessing intervention elements. Step 2: Assessing applicability issues in the local setting. Step 3: Resolving these issues with both intervention developers and the local practitioners. Moreover, we positioned these steps in a joint decision-making process in which researchers and local health care professionals were approached alternately. By combining the adaptation frameworks from existing literature with a practical decision model, we were able to gain insight into the actual adaptations of the intervention, the decisions and their justification. For this study, we obtained ethical approval of the Medical Ethical Committee of Wageningen University. Data were collected by a combination of desk research, focus groups and (e)mail correspondence.

### Local setting

The real-life setting selected for this study was the municipality of Apeldoorn, an average, middle-sized Dutch city (population 156,000). Apeldoorn has 56.648 inhabitants aged 40–65 years, of which 12% is of non-Dutch origin, the latter group consisting mainly of Western immigrants such as Germans (Central Bureau of Statistics, CBS). Based on a large survey conducted by the community health service (GGD Gelre-IJssel) in autumn 2008, 2% of the inhabitants aged 35–50 years and 7% of inhabitants aged 50–65 years report a diagnosis of diabetes. In addition, 1% reports impaired glucose tolerance and 46% were overweight based on self-reported weight and height. Apeldoorn has about 80 general medical practices, most of which are organised as solo- or duo practices. About a quarter are organised as group-practices. Additionally, there are nine Health Care Centres where general practitioners work together with other health care providers such as dieticians and physiotherapists. There are about 40 physiotherapist practices in Apeldoorn. Almost all dieticians (approximately 20 practices) in Apeldoorn are employed by the Home Care organisation Vérian; only 6 have their own businesses.

In order to develop a basis for collaboration between the real-life setting and the researchers, a local steering committee was established in November 2008. The steering committee was presided by the Community Health Service – a Dutch regional public health service with academic expertise as well as sound knowledge of the local structures. Other partners in the local steering committee were the university, the local authorities, the regional supporting organisation for primary health care and the local organisation of general practitioners (diabetes care group). Later on, the steering committee was completed with two representatives, one for the local dieticians and one for the local physiotherapists (April 2009). The multidisciplinary character of the local steering committee was considered essential in order to bridge the gap between prevention and primary health care. The task of the local steering committee was to facilitate the decision process wherever needed.

### Participants

A total of 16 health care professionals was invited: four general practitioners and their practice nurses, five physiotherapists and three dieticians. They were selected by discussion and consensus among the steering committee, with the main criteria being enthusiasm and local leadership in the field of diabetes prevention. It was ensured that the different types of organisation and different neighbourhoods of Apeldoorn were represented. As well as this, two out of the three SLIM developers who had been involved from the early stages were approached (among which EJMF). These two were selected by the committee for the practical reason of geographical distance. The committee also selected a health promotion expert equipped for implementation issues (JTB). The health promotion expert was added to the panel for the competencies in public health and prevention, which was seen as relevant and complementary to the input of the primary health care professionals. The panel was invited for a collective meeting in the city hall of Apeldoorn, where the procedure was explained (November 2009). They were asked to agree to both the procedure and the requested time investment.

### Steps in the adaptation process

#### Assessing intervention elements

In the first step, the intervention elements of SLIM were identified. There is no clear definition of an intervention ‘element’ (or component) in the literature. For the scope of this article we defined ‘element’ as ‘a characteristic of an intervention which defines its nature, categorised into target population, techniques and instruments, intensity, delivery mode, materials, organisational structure, and political and financial conditions’, based on a combination of existing literature [7,15,18]. In order to identify SLIM elements, the SLIM archive was analysed. It contained research protocols, participant brochures and scientific articles from the period 1999 to present. All the documents were studied in order to obtain a detailed overview of the SLIM intervention. Knowledge gaps were filled in by the SLIM developers during semi-structured interviews where notes were taken. Afterwards, the information on the entire SLIM intervention was structured according to the categories of elements presented above. The separate SLIM elements in each category were identified and described.

#### Assessing applicability issues in the local setting

In this step, the list of SLIM elements was separated in two parts. The first part contained the elements at the professional (micro) level: target population, techniques, intensity and delivery mode. This part of the list was sent to the local health care professionals. They were asked to judge each element on the list as applicable or not applicable in the real-life setting of Apeldoorn. If not applicable, they were asked to provide arguments and to suggest adaptations. The second part of the list contained the elements on the organisational (meso) and societal (macro) level: materials, organisational structure, and political and financial conditions. These elements were judged by the health promotion expert in collaboration with the local steering committee. During this step, data were collected as written comments via post and e-mail. When necessary, additional information was collected through telephone contact. In the analysis, an intervention element was qualified as inapplicable when at least one person judged the element as inapplicable.

#### Resolving these issues with both intervention developers and the local practitioners

First, the results of step 2 were presented to the SLIM developers. They were asked to judge the adaptations suggested by local professionals and health promotion expert as ‘will/will not influence intervention effectiveness’. If influence on intervention effectiveness was expected, they were asked for alternatives and/or compromises. As a result, the SLIM developers produced a list of proposed adaptations. The adaptations could either lie in adjusting the intervention to suit the local setting, or in making the local setting more receptive to the intervention. The SLIM developers were challenged to find a balance between adapting the SLIM protocol and adapting the real-life working procedures.

Thereafter, the adaptations proposed by the SLIM developers were judged by the local professionals. To this end, the local health care professionals were invited for focus group meetings according to profession (general practitioners and/or practice nurses, dieticians and physiotherapists). The focus groups were guided by a trained discussion leader and structured with a topic list. The basic questions in the focus groups were: Are the proposed adaptations feasible in your opinion? Can you implement this intervention in practice? Any objections were discussed until consensus was reached, which was recorded by taking notes. Afterwards, the notes were analysed by two people to identify any consequences for the adaptation of SLIM. Finally, the adaptations were incorporated in the new SLIMMER manual, a reference for health care professionals which was adapted as a result of this study. The manual was presented to participants during a collective meeting in the city hall of Apeldoorn (August 2010).

## Results

### Response

Both SLIM developers, the health promotion expert and 13 of 16 local professionals accepted the invitation to participate in the adaptation process. All of them attended the collective meeting in the city hall and agreed on the procedure and time investment. The panel was representative for their professional group in Apeldoorn in terms of geographic location and type of organisation. In the first step of assessing SLIM intervention elements, both SLIM developers participated (Table 2). In the second step, 11 out of 13 local health care professionals and the health promotion expert participated. In the third step, both SLIM developers participated. For the focus groups, the response differed per professional group: all physiotherapists and 2 out of 3 dieticians were present. The focus group with general practitioners was cancelled on their request; instead they responded by e-mail. During the whole adaptation process, general practitioners sometimes answered on behalf of their practice nurses and vice versa. All participants attended the final collective meeting in the city hall, where they received the definitive SLIMMER manuals, adapted as a result of this study.

**Table 2:** Number of participant type by adaptation step

	**Panel (n)**	**Step 1 (n)**	**Step 2 (n)**	**Step 3 (n)**
Intervention developers	2	2	n.a.	2
Health promotion expert	1	n.a.	1	1
General practitioner/practice nurse	6	n.a.	5	6 (e-mail)
Physiotherapist	4	n.a.	3	4
Dietician	3	n.a.	3	2

N.a.,not applicable (participant type not invited).

### Results of step 1: assessing intervention elements

As described in the methods section, the elements of the SLIM intervention were identified in the first adaptation step. Most elements could be identified by studying the SLIM archive. However, protocols or instructions for the exercise training were lacking, and were filled in by the SLIM developers during interviews. The SLIM intervention was based on a mixture of theories, which were generally applied at that time (1999–2005). This included the Stages of Change model (Prochaska), the Theory of Planned Behaviour (Azjen), and behaviour change techniques such as motivational interviewing and goal setting. Only techniques that were explicitly mentioned in the archive were included in the list of SLIM elements. The final list contained a total of 22 elements: 16 SLIM elements in the categories target population, techniques, intensity and delivery mode, and six SLIM elements in the categories materials, organisational structure, and political and financial conditions (Table 3, first column).

**Table 3:** **SLIM elements identified** (**step 1**), **including applicability** (**step 2**) **and subsequent adaptations** (**step 3**)

	**Step 1**	**Step 2**		**Step 3**	
	**SLIM elements identified**	**Applicability as judged by local health care professionals**		**Adaptations proposed by SLIM developers and accepted by local health care professionals**	
		**Applicable Yes / No**	**Argument**	**Adaptation of SLIM protocol**	**Adaptation of real-life working procedures**
	**Target population**				
1	Selection from study cohort	N	GP is the location of finding high-risk groups for diabetes	selection by GP	GP actively search their database for patients with IFG and refer them to intervention
2	Selection with OGTT	N	OGTT not used	selection with FBGM	
3	Only Caucasian subjects	N	ethnicity of patients not known	only Dutch speaking subjects	
	**Techniques and instruments**				
4	Appropriate risk communication to participants	Y			
5	Dietary advice: motivational interviewing	Y			
6	Dietary advice: goal setting	Y			
7	Dietary advice: invite partner	Y			
8	Dietary advice: fixed theme per visit	N	themes are tailored to patient	order of themes may be changed	all themes should be addressed
9	Dietary intake with 3-day food record	N	- great variability in intake procedures	- no standard dietary intake	
			- no / simple nutritional diaries	- nutritional diaries not obliged	
10	Exercise training tailored to middle-aged people with overweight	Y			
11	Exercise intake with maximal test	N	- great variability in intake procedures	exercise intake with submaximal test (steep ramp)	standard use of steep ramp test during intake
			- maximal tests require medical supervision		
	**Delivery mode**				
12	Dietary advice: individual, group meeting once a year	Y			
13	Exercise training in groups of 4-6	Y			
14	Exercise training in special SLIM groups	N	creating separate groups is costly		organise special SLIMMER groups
	**Intensity**				
15	Dietary advice	N	Frequency and duration are	- decreasing time intervals between visits	no tailoring of frequency and duration to patient
	- every 3 months		tailored to patient		
	- duration 60 minutes		- intervals ≤ 2 months	- duration 30 minutes	
	- group meeting 90 minutes		- duration 15-30 minutes		
16	Exercise trainings	Y			
	- 1-2 times a week				
	- duration 60 minutes				
	**Step 1**	**Step 2**		**Step 3**	
	**SLIM elements identified**	**Applicability as judged by health promotion expert / local steering comittee**		**Adaptations proposed by SLIM developers and accepted by local health care professionals**	
		**Applicable Yes / No**	**Argument**	**Adaptation of SLIM protocol**	**Adaptation of real-life working procedures**
	**Materials**				
17	Patient brochures	N	black-and-white, text-only documents	up-to-date patient brochures from national institutes	
18	Manuals	N	- incomplete manuals	- manual developed for exercise training	
			- scientific language		
			- no distinction between intervention and research	- manuals in readable language, tailored to local professionals	
	**Organisational structure**				
19	Intervention deliverers are employed by the university; local organisations are not involved	N	Intervention delivery is complex cooperative process between local organisations	- roles and responsibilities described	
				- information meeting added to facilitate collaboration	
	**Political and financial conditions**				
20	Intervention embedded in national policy	Y			
21	Intervention embedded in local policy	Y			
22	Research subsidies	N	- structural finances needed	(not fulfilled)	(not fulfilled)
			- no natural financer		

FBGM, Fasting Blood Glucose Measurement. GP, general practitioner. IFG, Impaired Fasting Glucose. OGTT, Oral Glucose Tolerance Test.

### Results of step 2 and 3: assessing applicability issues in the local setting and resolving these issues with both intervention developers and the local practitioners

Out of 16 intervention elements, eight were judged as inapplicable by one or more local health care professionals. These concerned the categories target population, techniques, intensity and delivery mode. Moreover, four out of six intervention elements in the categories materials, organisational structure, and political and financial conditions were judged as inapplicable by the health promotion expert in collaboration with the local steering committee (Table 3, second and third column). The inapplicable elements that were most prominent or yielded severe discussion with the SLIM developers are described below.

#### Target population

The target population for SLIM was selected with the Oral Glucose Tolerance Test (OGTT); however local general practitioners judged the OGTT as inapplicable in the real-life setting. They suggested selecting participants with Fasting Blood Glucose Measurement (FBGM). This was confirmed by other authoritative sources of information: national working standards for Dutch primary and clinical care also recommend FBGM as the OGTT is costly, inconvenient and not very well reproducible [29]. In response, the SLIM developers emphasised that by using FBGM instead of OGTT, a different target group would be selected. This might influence the effectiveness of SLIM to an unknown extent. It is not completely clear from scientific literature whether lifestyle interventions such as SLIM are as beneficial for patients with Impaired Fasting Glucose (selected with FBGM) as for patients with Impaired Glucose Tolerance (selected with OGTT). However, finally the SLIM developers agreed that the necessary fit with the real-life setting should receive priority and it was proposed to change the selection procedure.

#### Techniques and instruments

Two of the techniques and instruments for dietary advice used in SLIM were judged inapplicable by local dieticians. The first was using extensive 3-day food records in the intake procedure. This type of diary is common in nutritional research; however in a real-life setting, intake procedures vary greatly and nutritional diaries are not used or only in simple formats. Introducing extensive 3-day food records would imply a substantial alteration of working procedures. Consultation with the SLIM developers made clear that these diaries were not considered essential for achieving effects; therefore it was proposed to drop these. Secondly, for each dietary visit in SLIM a fixed theme was given (e.g. carbohydrates, how to deal with parties, how to read nutritional labels). However local dieticians reported not to work with fixed themes, but to tailor themes to patients. Consultation with the SLIM developers revealed that the selected themes (but not their order) were considered essential for knowledge transfer and subsequent behaviour change. It was therefore proposed that dieticians could change the order of themes as long as all themes were discussed.

#### Intensity

The intensity of dietary advice as reported in SLIM the protocol was judged as inapplicable by local dieticians. Dietary visits in SLIM were scheduled every 3 months with a duration of 60 minutes, and an annual group session of 90 minutes. Local dieticians reported to work with shorter and more frequent visits (intervals ≤ 2 months and duration 15–30 minutes). Moreover, they reported to tailor the frequency and length of visits to their patients, and some of them were not used to organising group sessions. Consultation with SLIM developers made clear that the group session and the total contact-time per patient per year was perceived essential for effectiveness, but not the exact frequency and duration of visits. Consequently, it was proposed to rearrange the schedule of visits (see Table 3); the group session of 90 minutes remained unchanged.

#### Delivery mode

The exercise training was delivered in special SLIM groups, which was judged inapplicable by local physiotherapists. It was argued that arranging such groups was not attractive from a cost perspective. Instead local physiotherapists suggested allocating subjects to existing medical training groups. Consultation with the SLIM developers however, made clear that the special SLIM groups were believed to be a key factor for success. In SLIM the group cohesion was very strong and continued outside the programme, e.g. participants spontaneously organised cooking and walking events. Therefore the SLIM developers insisted on retaining the special exercise groups.

#### Materials

The health promotion expert judged the SLIM materials as inapplicable for the real-life setting. The patient brochures were mostly black-and-white text-only documents, whereas in practice attractive brochures are needed, tailored to the target group. It was decided to replace the patient brochures with up-to-date brochures from official national institutes (Dutch Centre for Nutrition and Dutch Heart Foundation). The manuals for intervention deliverers were incomplete (no manual available for exercise training), used scientific language, and made no distinction between intervention delivery and research measurements. It was decided to rewrite the manuals in readable language, tailored to local health care professionals, and to develop a manual for the exercise training.

#### Organisational structure

The health promotion expert noticed that the organisational structure in SLIM was not comparable to the real-life setting. The SLIM intervention deliverers were directly employed by the university; whereas in real-life, the intervention is delivered by regular health care personnel from a several local organisations. This is a complex cooperative process; which requires that roles and responsibilities are clear and that interaction between professionals is facilitated. Therefore, roles and responsibilities between local organisations were discussed on the management level (in the local steering committee) and a paragraph on this topic was added to the manual. Moreover, an information meeting was added in the first phase of the intervention. The general practitioner, dietician and physiotherapist were asked to collectively organise this meeting for patients in their neighbourhood. The principal aim of the information meeting was to introduce patients to the professionals involved in the intervention, and motivate patients to participate. In addition, the implicit aim of the information meeting was to stimulate interaction and team spirit among local health care professionals, by providing them with a common task.

#### Political and financial conditions

The health promotion expert noticed that the SLIM intervention fitted well into the Dutch national health priorities of that time [30]. However, for such an intervention to be successful in a real-life setting, it should also fit in with local policies. In Apeldoorn, this condition was fulfilled since the local authorities, the local organisation of general practitioners (diabetes care group) and the regional supporting organisation of primary health care considered diabetes prevention as a priority. However, the financial conditions were not fulfilled. Since SLIM is an intervention between prevention and primary health care, neither the local authorities nor the health care insurer were natural financers. So far, we did not succeed in finding structural finances for the implementation of SLIM in the real-life setting, despite several attempts. Other authoritative sources of information confirmed that the Dutch financing and legislation systems for prevention and primary health care are not closely linked, which makes the implementation of interventions such as SLIM very difficult.

#### Proposed adaptations

In Table 3 (fourth and fifth column) the proposed adaptations for target population, techniques, intensity, delivery mode, materials, organisational structure, and political and financial conditions as described above are presented. In summary, for 6 out of 12 inapplicable intervention elements the SLIM protocol was adapted to suit real-life working procedures. For 1 out of 12 inapplicable intervention elements, the working procedures in the real-life setting were adapted based on the SLIM protocol. For 4 out of 12 intervention elements, a compromise was found. And finally, for 1 out of 12 inapplicable intervention elements no solution could be found at all (financial conditions). The proposed adaptations were incorporated in the new SLIMMER manual.

### Focus group results

During the focus groups, local health care professionals discussed the new SLIMMER manual, in which all proposed adaptations as described above were incorporated. Generally, the SLIMMER manual was judged to be of good quality. However, four proposed adaptations were initially judged as unfeasible. In the physiotherapist group, the discussion concentrated on ‘standard use of Steep Ramp Test during intake’ and ‘organise special SLIMMER groups’. There was resistance among physiotherapists to accept top-down standard procedures. By discussing the importance of unity in working procedures and explaining the financial compensation, the resistance diminished more and more. In the dietician group, there was low self-confidence for ‘Organising group meetings’ and ‘Working with goal setting’. The self-confidence was strengthened by articulating faith in the abilities of the dieticians and discussing how they already use these techniques in daily practice. At the end of the focus groups, all discussions could be finalised towards consensus and all adaptations were judged as feasible.

## Discussion

The aim of this study was to describe the process of adapting evidence-based interventions to real-life settings, using the SLIM diabetes prevention intervention as an example. We adapted the SLIM intervention and developed the SLIMMER intervention, which is applicable in the Dutch real-life setting. Three adaptation steps were used, according to current adaptation frameworks, in order to facilitate the joint decision making process of SLIM developers and local health care professionals. During the first step 22 SLIM elements were identified. During the second step, 12 of these 22 elements were judged inapplicable by the local health care professionals or health promotion expert. In the third step, adaptations for these 12 elements were proposed by the SLIM developers, and discussed and finally accepted by all participants. The positive result of this study is that consensus was achieved between SLIM developers and local health care professionals on the adaptations needed to make SLIM applicable in a Dutch real-life setting within a relatively short time period (nine months). Our study shows that it is possible to unite the perspectives of scientists and practitioners, finding a balance between evidence-base and applicability concerns.

There may be some limitations to our study. To begin with, we used a heterogeneous panel of 16 participants representing the different professional groups, to ensure optimal variation in the expertise needed for our study. For a heterogeneous panel, the group was relatively small, which makes it difficult to conclude whether or not saturation of arguments was reached. However, there are no indications that a larger panel would have lead to other adaptation decisions. Besides, the panel was localised, since our aim was to adapt SLIMMER to the local real-life setting. The disadvantage of a localised panel may be that the adaptations are only valid in the local setting under study and cannot be generalized to other settings. However, we raised the chances for successful implementation across other local settings by verifying the opinion of local health care providers with other authoritative sources, such as national institutes and national guidelines. Based on these comparisons, we know that the adaptations made to SLIM correspond with national health care practices.

To conclude, the change in process from an in-person focus group to e-mail responses by general practitioners could be a limitation. First, it could be a limitation of engagement. We incorporated in-person focus groups in the adaptation process in order to stimulate engagement of local health care professionals. However for general practitioners this did not work; the idea of a time-consuming focus group rather lowered their engagement. By allowing e-mail responses we were able to keep them involved. Moreover, there was only one adaptation that directly concerned general practitioners. This point was prepared orally with one of the general practitioners and thereafter accepted by all general practitioners via e-mail responses. We do not expect that a focus group would have yielded a different result here.

As indicated by Backer [15] one of the first steps of the adaptation process should be to identify the theory behind the intervention and to indicate its core elements. Ideally the ‘core’ elements should be maintained during the adaptation process, in order to preserve intervention effectiveness. Core elements are defined as those elements of an intervention that fundamentally define its nature and account for the intervention effects. However the problem is that very few evidence-based interventions have evidence on core elements available [12]. In the absence of such research evidence for SLIM, we relied on the perception of intervention developers for the expected influence on intervention effectiveness. The advantage of this approach was that the process of adaptation could be finished within a relatively short time period (nine months), which kept local health care professionals engaged during the whole process. However, this information is subjective and should be complemented with an intervention theory or intervention logic model in order to elucidate core elements [13,15]. Currently, further research into the core elements of the SLIMMER intervention is being conducted making use of a taxonomy to identify behaviour change techniques [31]. This will allow us to re-construct the intervention logic model of SLIMMER and evaluate the mechanism of change during the next steps of our study.

One of the strengths of this study was the successful collaboration in the local steering committee, which was established as a first step before the adaptation process was started. We feel that several factors have contributed to this success, including the fact that the diabetes care group in Apeldoorn is one of the first in the Netherlands; it was already mature and stable at the time which broadened the perspective for prevention; the experience and personal engagement of all partners, and the fact that enough time was taken for starting-up the local steering committee (a full year) in order to build confidence.

Our study showed that it is possible to adapt an evidence-based intervention in such a way that it is feasible for the relevant partners in a local setting, finding a balance between evidence-base and applicability concerns. The next steps in the adaptation process, as indicated by McKleroy [14], are the pilot-testing of the adapted intervention, which may lead to further refinement, followed by an evaluation of the cost-effectiveness of the adapted intervention and its final implementation. These steps are being undertaken at the moment in further research on SLIMMER.

## Conclusions

This study provided an example of the process for the adaptation of an evidence-based intervention to a real-life setting. We used an adaptation process of 3 steps with joint decision making between the intervention developers and local health care professionals, and adapted the SLIM diabetes prevention intervention to a Dutch real-life setting. Initially 12 out of 22 intervention elements were judged as inapplicable in the local setting, but they were successfully adapted with consensus from both researchers and practitioners. The adapted elements concerned the categories target population, techniques, intensity, delivery mode, materials, organisational structure, political and financial conditions. The adaptations either lay in changing the SLIM protocol (6 elements) or the real-life working procedures (1 element), or both (4 elements). Our study shows that it is possible to unite the perspectives of scientists and practitioners in a joint decision making process, thus finding a balance between evidence-base and applicability concerns.

## Competing interests

The authors declare that they have no competing interests.

## Authors’ contributions

SCJ conceived the study design, collected and processed all data, and drafted the manuscript. AH contributed to the study design and made major revisions to the manuscript. GD participated in the study design and data collection. JTB participated in the scoring of intervention elements and data processing. GJH and EJMF participated in data interpretation and helped to draft the manuscript. All authors read and approved the final manuscript.

## Pre-publication history

The pre-publication history for this paper can be accessed here:

http://www.biomedcentral.com/1471-2458/13/457/prepub
